# The Relationship Between Social Integration and Physical Activity, Diet, and Sleep Among Youths: Cross-sectional Survey Study

**DOI:** 10.2196/40354

**Published:** 2022-11-23

**Authors:** Bridget Wray, Amanda Grimes, Katlyn Eighmy, Joseph Lightner

**Affiliations:** 1 School of Nursing and Health Studies University of Missouri-Kansas City Kansas City, MO United States; 2 School of Public Affairs and Administration University of Kansas Lawrence, KS United States; 3 Department of Population Health University of Kansas Medical Center Kansas City, KS United States

**Keywords:** social integration, youth, nutrition, sleep, physical activity, adults, exercise, health, wellness, health behavior, school students, diet, children, health behavior intervention

## Abstract

**Background:**

Social integration has been shown to predict physical activity (PA), diet, and sleep in adults. However, these associations have not been well-studied in youth samples. Using a life course perspective, it is imperative to study this in youths as social and health behaviors are established early in life.

**Objective:**

The purpose of this study was to understand the relationship between social integration and PA, diet, and sleep for urban, middle-school youth.

**Methods:**

Cross-sectional baseline data from middle-school youths (N=73) who participated in an afterschool health behavior intervention were included in this study.

**Results:**

Time with friends significantly predicted moderate to vigorous intensity PA (β=.33, *P*=.02). Time spent with family was significantly related to fruit consumption (*t*_66_=1.38, *P*=.005) and vegetable consumption (*t*_72_=1.96, *P*=.01).

**Conclusions:**

Social integration appears to be related to both PA and nutrition behaviors in youths. Future research should expand on our findings to explain how different domains of social integration may impact youths’ health behaviors.

**International Registered Report Identifier (IRRID):**

RR2-10.2196/37126

## Introduction

The Centers for Disease Control and Prevention recommends healthy eating, physical activity (PA), and optimal sleep for youths to achieve and maintain a healthy weight [1]. Youths who meet recommendations for PA, diet, and sleep are more likely to display healthy growth, body composition, physical fitness, cognitive development, academic achievement, and overall quality of life [2], in addition to a decreased mortality risk later in life [3]. Unfortunately, the majority of youths do not meet these recommendations for PA [4], diet-related behaviors [5], or sleep [6]. Youths of color, especially females of color, are more likely to experience physical inactivity, a poor diet, and poor sleep patterns [7].

Nearly 6 in 10 youths lack any PA outside of school settings, and PA typically declines as youths age [8]. Similarly, youths develop food preferences from their childhood and are often maintained throughout life [9]. These preferences can promote or hinder, youths’ healthy eating habits into adulthood [9]. Sleep duration for youths tends to be less than optimal and continues to decrease with age, resulting in a negative impact on school performance, emotional health, and physical health [10]. Improving health behaviors early in life is essential to improving population health, as youths establish patterns early in life that extend into adulthood [11]. One factor influencing youths’ health-promoting behaviors is their social environment. The social environment is an accumulation of many aspects of social life, including social networks, the social support of those in the network, and social integration. More specifically, evidence suggests that social integration may have a unique influence on behaviors that are important to healthy development and disease prevention. Social integration is the interaction between an individual and their social environment, including both formal (eg, participation in sports clubs and church) and informal aspects (eg, spending time with family and friends) [12].

Informal social integration has been shown to be related to health. For example, social integration influences PA, both cross-sectionally and longitudinally, where informal social integration with friends appears to be more predictive of PA than informal social integration with family [13]. Informal integration also predicts higher fruit and vegetable consumption [14]. Those who are more socially integrated have higher-quality sleep than those who are less socially integrated [15]. Overall, social integration has been linked with a reduced mortality risk [16,17].

While evidence is well-developed in adult populations, the relationship between social integration and PA has not been well-studied in youths, especially among racially and ethnically marginalized adolescents traditionally underrepresented in research. From a life course perspective, it is imperative to study this in youths, as social and health behaviors are established early in life. Therefore, the purpose of this study is to examine and describe the relationship between social integration and PA, nutrition, and sleep behaviors among urban, middle-school youths.

## Methods

### Overview

This study used baseline data from an after-school program to improve health behaviors in middle-school youths (ages 10-14). Parents and youths provided written informed consent and assent, respectively, before participating in this study. The after-school program provided web-based opportunities for exercise sessions such as yoga, dance, and general cardio endurance exercise owing to COVID-19. Fresh produce was available for pick-up once per week or delivered to families if they lacked transportation.

Middle-school youths in the Kansas City Public School District, Kansas City, Missouri, participated in this study. Of the 14,128 students in the district, 54% are Black, 27% are Hispanic or Latinx, 11% are White, and 8% are of other races and ethnicities [18]. All students in Kansas City Public Schools qualify for free school breakfast and lunch.

The data were collected between August 2020 and May 2021. As part of baseline data collection, youths completed a survey that assessed obesogenic behaviors, including PA, social integration, dietary behaviors, and sleep. Youths (n=76) who completed a baseline survey represented sixth, seventh, and eighth graders. Those who did not provide any valid answers to the nutrition, sleep, PA, or social integration questions were excluded (n=3), resulting in a sample of 73 youths.

### Ethics Approval

All study procedures were approved by the institutional review board of the University of Missouri-Kansas City under protocol number #2017528.

### Measures

#### PA

Self-report measures of PA were collected using the International Physical Activity Questionnaire (IPAQ) Short Form [19]. The IPAQ Short Form is a 7-day recall questionnaire to estimate recent PA behavior and was used to calculate vigorous- and moderate-intensity PA [19]. Youths reported time spent doing moderate-intensity activities in the past 7 days if the activity required moderate physical effort and breathing, such as carrying light loads, bicycling at a regular pace, or doubles tennis. Youths reported time spent doing vigorous-intensity activities in the past 7 days if the activity required hard physical effort and breathing, such as heavy lifting, aerobics, or fast bicycling. The IPAQ Short Form also measures walking and sedentary behavior. However, time spent walking and sitting is not presented in this study. Three responses were 3 SDs away from the mean and excluded from data analysis for being outliers.

#### Social Integration

Youths’ social integration was measured through an adapted version of Cundiff and Matthew’s 1-item measure [20]. The question was adapted into a 2-item measure for youths to report separately on time spent with friends and family. Response options were reported on a 5-point scale that ranged from <1 hour per week to >20 hours per week for time with family and time with friends, respectively.

#### Dietary Behaviors

Dietary questions were adapted from the 2019 Youth Risk Behavior Survey High School instrument to measure dietary behaviors [21]. Questions were asked about fruit, vegetable, soda, and sports drink consumption in the past 7 days. The original Youth Risk Behavior Survey response options ranged on a 7-point scale from no consumption to >4 times a day; for this study, these response options were collapsed into 3 response categories (*yes*, *no*, and *not sure*). *Not sure* responses were excluded from data analysis.

#### Sleep

Sleep was measured by a single question from the 11-item Kutcher Adolescent Depression Scale [22]. Youths reported sleep difficulties from the past 7 days, with response options ranging from *hardly ever*, *much of the time*, *most of the time*, to *all of the time*. *Hardly ever* responses were classified as no persistent sleep issues. *Much of the time*, *most of the time*, and *all of the time* responses were combined and classified as having sleep issues. Sleep was collapsed into a dichotomous variable (0=no sleep issues, 1=persistent sleep issues) for data analysis.

### Analysis Plan

Univariate statistical analyses were conducted for all study variables. Independent samples *t* tests were conducted to examine differences between dichotomous variables (diet and sleep) and social integration variables. Linear regression analyses were used to estimate the effect of social integration on moderate to vigorous intensity PA (MVPA). All statistical analyses were conducted using SPSS software (version 26; IBM Corp) [23].

## Results

Youths’ demographics are presented in Table 1. Among the participating youths (N=73), 34 (47%) identified as female and 38 (52%) identified as male. The sample diversely represented race/ethnicity groups, with 44% (32/73) reported being African American or Black, 26% (19/73) reported being White, 6% (4/73) reported being Hispanic` or Latinx, 7% (5/73) reported being Asian, and 18% (13/73) reported being multiracial or multiethnic. All youths were in the sixth, seventh, or eighth grades (mean age 12.04, SD 0.93 years). Regarding youths’ diet and sleep behaviors in the last 7 days, 61% (40/66) reported eating fresh fruit, 61% (43/70) reported eating vegetables, 26% (19/72) reported drinking soda, 16% (11/69) reported drinking a sports drink, and 51% (36/71) reported abnormal sleep issues. Youths reported engaging in 286.40 (SD 307.19) minutes of MVPA per week. Youths reported social integration on a 5-point scale. Youths reported time with family at 4.26 (SD 1.21) or between 11 to 20 hours per week and time with friends at 2.02 (SD 1.12) or between 1 to 5 hours per week.

Tables 2 and 3 present the results of the *t* tests that examined differences in social integration for fruit, vegetable, soda, and sports drink consumption and sleep difficulties. Fruit consumption was significantly related to time spent with family (*t*_66_=1.38, *P=*.005) but not significantly related to time spent with friends (*t*_66_=2.61, *P*=.08). Vegetable consumption was significantly related to time spent with family (*t*_72_=1.96, *P*=.012) but not significantly related to time spent with friends (*t*_72_=0.067, *P*=.68). Youths who spent more time with family were more likely to consume fruit and vegetables on the last day. There were no substantial differences in time spent with family or friends based on soda, sports drinks, and sleep variables.

Table 4 presents the results of the linear regression analyses examining associations between social integration and PA. MVPA was related to time spent with friends (β=.33, *P*=.02) but not to time spent with family (β=.01, *P*=.94). For every 1 SD increase in time spent with friends, MVPA increased 0.33 SDs.

**Table 1:** Univariate statistics.

Variables			Values
**Sex, n (%)**			
	Female	34 (47)	
	Male	38 (52)	
	No response	1 (1)	
Age (years), mean (SD)			12.04 (0.9)
**Race and ethnicity, n (%)**			
	African American or Black	32 (44%)	
	White	19 (26%)	
	Asian	5 (7%)	
	Hispanic or Latinx	4 (6%)	
	Multiracial or multiethnic	13 (18%)	
**Fresh fruit consumption, n (%)**			
	Yes	40 (61%)	
	No	26 (39%)	
**Vegetable consumption, n (%)**			
	Yes	43 (61%)	
	No	27 (39%)	
**Soda consumption, n (%)**			
	Yes	19 (26%)	
	No	53 (74%)	
**Sports drink consumption, n (%)**			
	Yes	11 (16%)	
	No	58 (84%)	
**Sleep issues, n (%)**			
	Yes	36 (51%)	
	No	35 (49%)	
MVPA^a^ (minutes per week), mean (SD)			286.4 (307.2)
Time with family			4.3 (1.2)
Time with friends			2.0 (1.1)

^a^MVPA: moderate to vigorous intensity physical activity.

**Table 2:** Associations between social integration and dietary behaviors.

Variable			Consumed, mean (SD)		Did not consume, mean (SD)		*t* test (*df*)		*P* value
**Fruit**									
	Time with family	4.44 (1.03)		4.00 (1.41)		1.38 (66)		.005	
	Time with friend	2.34 (1.25)		1.57 (0.84)		2.61 (66)		.08	
**Vegetable**									
	Time with family	4.49 (1.07)		3.87 (1.39)		1.96 (70)		.012	
	Time with friend	2.12 (1.17)		1.91 (1.08)		0.67 (70)		.68	
**Soda**									
	Time with family	4.07 (1.39)		4.33 (1.18)		–0.71 (72)		.12	
	Time with friend	2.00 (1.04)		2.05 (1.16)		–0.13 (72)		.81	
**Sports drink**									
	Time with family	4.30 (1.25)		4.27 (1.22)		0.06 (69)		.95	
	Time with friend	2.20 (1.03)		2.07 (1.16)		0.34 (69)		.93	

**Table 3:** Association between social integration and sleep behaviors.

Variables	No persistent sleep issues, mean (SD)	Persistent sleep issues, mean (SD)	*t* test (*df*)	*P* value
Time with family	4.21 (1.27)	4.31 (1.18)	0.33 (71)	.54
Time with friend	2.13 (1.17)	1.90 (1.08)	–0.81 (71)	.76

**Table 4:** Associations between social integration and moderate to vigorous intensity physical activity.

Variable	β	SE	95% CI	*P* value
Time with family	.01	32.90	–67.84 to 72.44	.94
Time with friends	.33	35.62	13.96 to 157.12	.02

## Discussion

The purpose of this study was to understand associations between social integration and PA, diet, and sleep behaviors for a sample of urban, middle-school youths. Overall, this study found that social integration is a substantial predictor of PA and fruit and vegetable consumption for middle schoolers in Kansas City, Missouri. However, social integration did not appear to be associated with other diet-related behaviors or sleep.

Our findings suggest that time spent with friends could be an essential component of youths’ PA, as time spent with friends was associated with MVPA. These results are consistent with previous findings in adult samples; time spent with friends significantly predicted PA both cross-sectionally and longitudinally [13,24]. Our results also align with those of previous research that found peer influence and socialization to be the most commonly cited motivators for PA among middle schoolers [25]. Spending time with family appears to have less impact on youths’ PA, similar to adult samples where time spent with family did not predict or had weaker associations with PA [13,24]. Despite these findings, previous research does indicate that parental involvement can increase youths’ PA [26]. The results of this study support the growing body of evidence that different domains of social integration, specifically time with friends, can be a powerful predictor of PA in youths.

In this sample, time spent with family was related to fruit and vegetable consumption, but time spent with friends was not. Our findings align with those of similar studies in adults, where social integration was found to be a substantial predictor of fruit and vegetable consumption [14]. Our results suggest that social integration, specifically time with family, appears to be a large predictor of diet behaviors in youths. Future studies should be conducted with larger samples, different populations, and other ages to understand if the results of this study are consistent across varying groups.

Social integration was not shown to be a predictor of improved sleep among youths in this study. These findings contradict those of similar studies performed with adult samples, which found that adults with higher social integration had higher sleep quality [15]. Our findings may be impacted by a notable increase in sleep disturbances during the COVID-19 pandemic in school-aged youths [27].

To our knowledge, this is the first study to assess the role of social integration with PA, diet, and sleep behaviors in youths. This study is strengthened by the participation of racially and ethnically marginalized youths. Current research consistently underrepresents populations of color [28,29]. Participation by underrepresented groups provides valuable insight into the specific associations between social integration and PA, diet, and sleep for youths in these groups. This study is also strengthened by the use of validated self-report measures.

This study is limited by its small sample size, potential self-report issues, and lack of generalizability to other populations. Self-reported data has several limitations, including social desirability bias, overestimation of behavior, and poor recall. Future studies should attempt to use measures that provide more variability and rigor (ie, a tool to measure multiple elements of sleep hygiene) and collect data using a monitoring device, such as accelerometry. This study also took place during the COVID-19 pandemic, which disrupted communication and contact with youths as school instruction transitioned to remote web-based learning. The pandemic may have impacted time spent with friends and family as well as PA, sleep, and diet behaviors. Further, we did no assess important physical environmental-level factors such as availability of parks or grocery stores, walkability of neighborhoods, community assets that may impact PA, diet, sleep, and social integration in these populations. Future research should consider attempting to understand how the social and physical environments interact to influence PA, diet, and sleep.

Our findings support the notion that different domains of social integration may promote positive health behaviors in youths. Increased time with friends may promote MVPA in youths. School sports, before- or after-school programs, or other group-based programs may offer a valuable opportunity to work with friend groups to increase PA. However, these types of programs may not be available to middle schoolers, have a fee to participate, or be competitive. These barriers may make sports and other programs unavailable to those who need it most [30]. Additional opportunities to increase youths’ social integration with friends to promote PA may include parental influence by fostering increased peer-to-peer engagement during nonschool hours. Increased time with family may promote increased fruit and vegetable consumption in youths, although families with low incomes and lower educational attainment are less likely to have accurate nutrition knowledge [31]. Therefore, increasing parent knowledge around nutrition is essential to transfer accurate nutrition knowledge and behavior to youths. Future research should investigate how these different aspects of social life may increase social integration and thereby influence PA and diet behavior.

## Abbreviations

IPAQ: International Physical Activity Questionnaire
MVPA: moderate-to-vigorous physical activity
PA: physical activity
